# Supplementary figures and images for: 5′-Modifications improve potency and efficacy of DNA donors for precision genome editing
Source: eLife. 2021 Oct 19;10:e72216. doi: 10.7554/eLife.72216 (PMC8568340; doi:10.7554/eLife.72216)

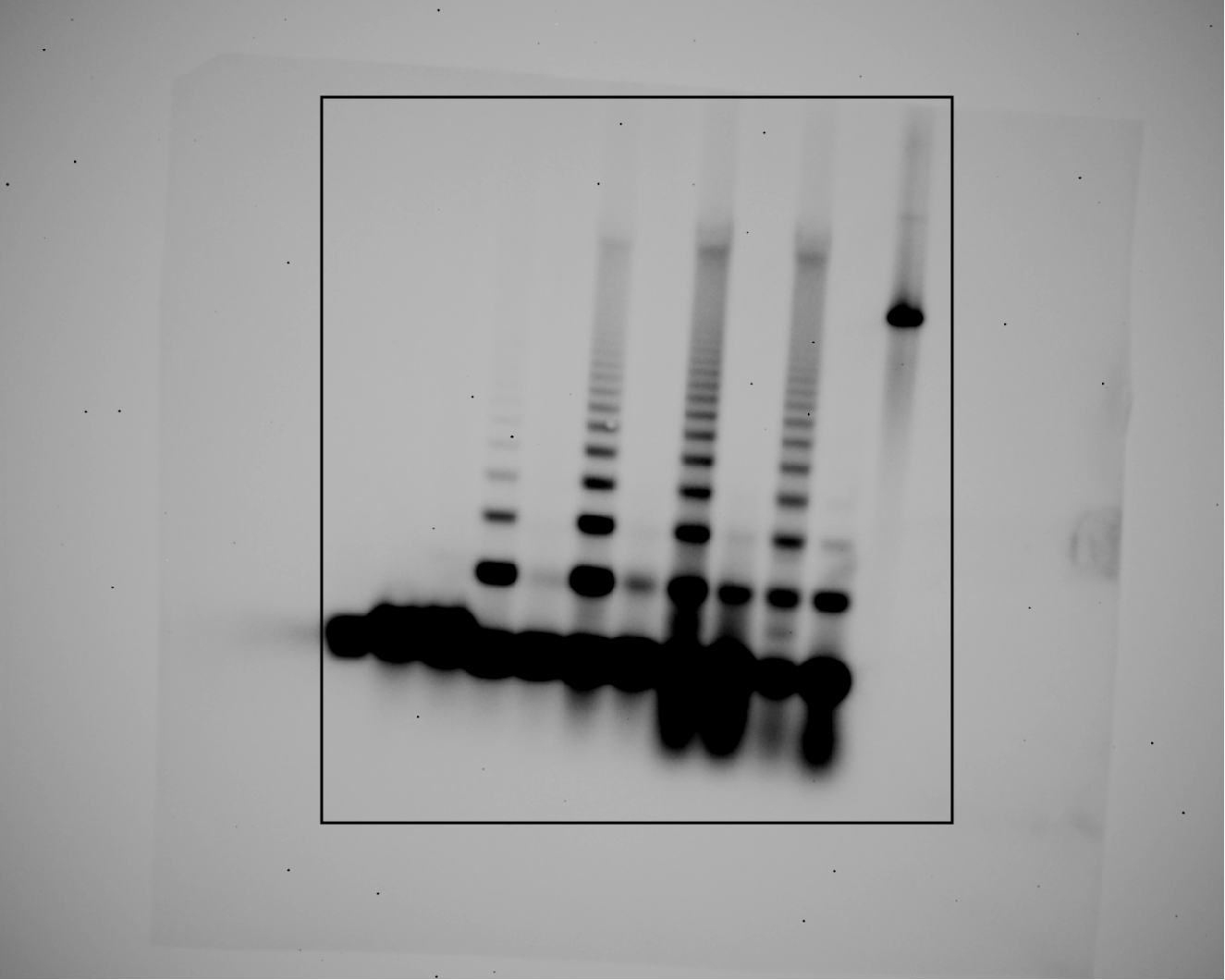

Supplement: Figure 6—source data 1. — Area in the box is shown in the main figure. [file elife-72216-fig6-data1.zip › Figure 6-source data 1.tif]

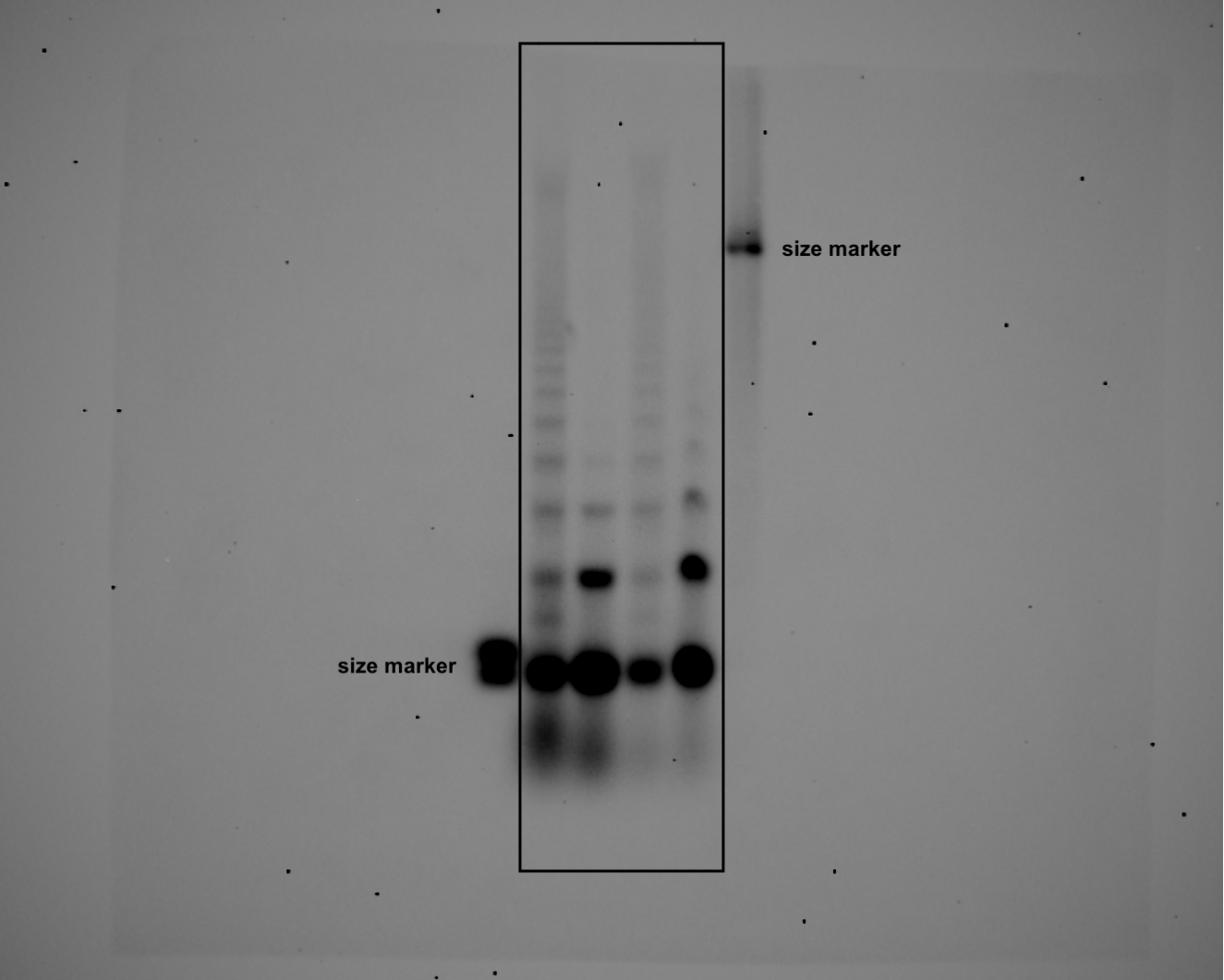

Supplement: Figure 6—source data 2. [file elife-72216-fig6-data2.zip › Figure 6-source data 2.tif]

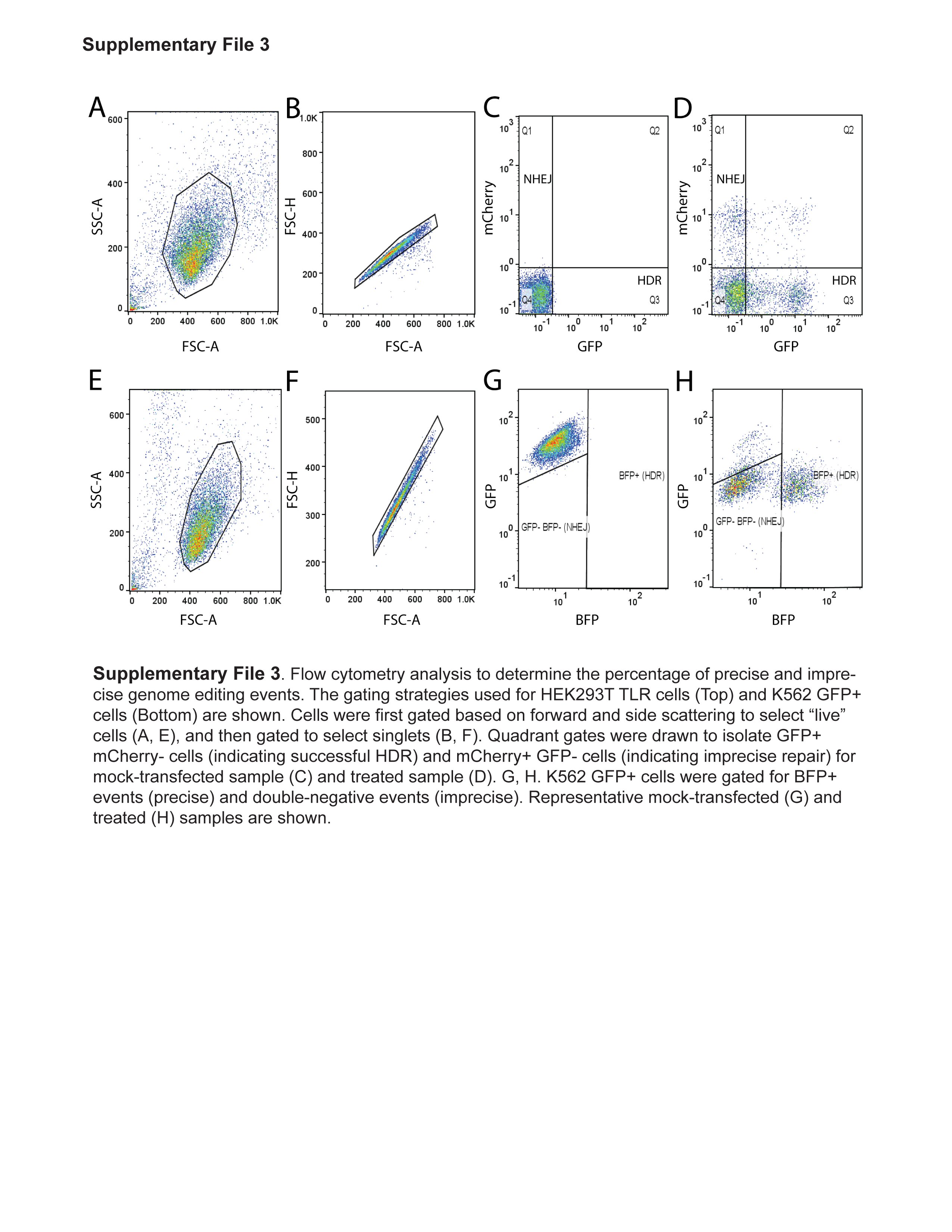

Supplement: Supplementary file 3. [file elife-72216-supp3.zip › Supplementary File 3.tif]
