# Supplementary material for: 5′-Modifications improve potency and efficacy of DNA donors for precision genome editing
Source: eLife. 2021 Oct 19;10:e72216. doi: 10.7554/eLife.72216 (PMC8568340; doi:10.7554/eLife.72216)
Supplement: Supplementary file 1. [file elife-72216-supp1.docx]

| **Supplementary File 1. Sequences of guide RNA spacers** | |  |
| --- | --- | --- |
|  |  |  |
| **Name** | **Guide sequence** | **Species** |
| Traffic Light Reporter_2.0 | GAGACAAATCACCTGCCTCG | *eGFP* |
| GAPDH | gagagagaccctcactgctg | *H.spaiens* |
| SEC61B-1 | CCCTCATCTCCAATATGGTA | *H.spaiens* |
| TOMM20 | AATTGTAAGTGCTCAGAGCT | *H.spaiens* |
| EMX1 | GAGTCCGAGCAGAAGAAGAA | *H.spaiens* |
| GFP-to-BFP | GCACTGCACGCCGTAGGTCA | *eGFP* |
| CMG-48 (*gfp*) | CCATCTAATTCAACAAGAAT | *C.elegans* |
| CMG-49*(gfp)* | CCTGAAAATTTAAATATGTA | *C.elegans* |
| CMG-67(gfp mutant(ne4807)) | GTTGTCCTGTTGTTAGTTAG | *C.elegans* |
| CMG-33(Flag::linker::tev) | TATAAAGACGATGACGATAA | *C.elegans* |
| 9209(*hey2* Lb-cpf1) | GTGTCTGTACCTGCGCGCACTGC | *D. rerio* |
| CMG-63 (*Sox2*) | TGCCCCTGTCGCACATGTGA | *Mus musculus* |
| CMG-89 (*Tyr*) | AACTGCGGAAACTCTAAGTT | *Mus musculus* |
