# Supplementary material for: 5′-Modifications improve potency and efficacy of DNA donors for precision genome editing
Source: eLife. 2021 Oct 19;10:e72216. doi: 10.7554/eLife.72216 (PMC8568340; doi:10.7554/eLife.72216)
Supplement: Supplementary file 2. [file elife-72216-supp2.docx]

| **Supplementary File 2. Sequences of oligos** | |
| --- | --- |
| **Structure of 2′Ome-RNA::TEG oligos** | 2’OMeRNA(GGAAGGGCCGAGCGC) – TEG spacer – DNA(oligo) |
| **Mammalian cell cultures** | |
| TLR2.0-donor-F | GGGCCAAGAACAGATGGTCA |
| TLR2.0-donor-R | GGCGGATCTTGAAGTTCACC |
| Sec61-B-donor-F | GGGCCCACACTAAAGTTAGAG |
| Sec61-B-donor-R | GCGCCATTGGGATGTTCAG |
| TOMM20-donor-F | GACGCGTATTGGGATGATGA |
| TOMM20-donor-R | GCGCCATTGGGATACCTTAA |
| GAPDH-donor-F | CTCCTGCACCACCAACT |
| GAPDH-donor-R | TGGGGTTACAGGCGTGC |
| GFP_to_BFP_donor | GTGCCCTGGCCCACCCTCGTGACCACCCTGTCTCATGGAGTTCAGTGCTTCAGCCGCTACCCCGAC |
| 05_emx1F_90bp | TGGCCCAGGTGAAGGTGTG |
| 06_emx1R_90bp | GGTTGCCCACCCTAGTCATT |
| 108_emx1_250hr_F | GCCCTGCCATCCCCTTCTGT |
| 109_emx1_250hr_R | CCATTGCTTGTCCCTCTGTC |
| 114_trac-250hr_F | GATAGCTTGTGCCTGTCCCT |
| 115_trac-250hr_R | AGAACCTGGCCATTCCTGAA |
| EMX1 PCR1_F_NGS | ctacacgacgctcttccgatctGGCCTCCTGAGTTTCTCATCT |
| EMX1 PCR1_R_NGS | agacgtgtgctcttccgatctCAGCACTCTGCCCTCGT |
| gSeq_F_guideseq | G*T*TTAATTGAGTTGTCATATGTTAATAACGGT*A*T |
| gSeq_R_guideseq | A*T*ACCGTTATTAACATATGACAACTCAATTAA*A*C |
| ***C. elegans*** | |
| cmo17659_csr1 donor | CGATTGGAAGTAGAGGTTCT |
| cmo17660_csr1 donor | ATCATGATATTGACTATAAA |
| cmo-KG686F_eft3-gfp-donor | ATGAGTAAAGGAGAAGAACT |
| cmo-KG687R_eft3_gfp_donor | TATCACCTTCAAACTTGACT |

| **Mouse** | |
| --- | --- |
| cmo_KG993F_tyr-donor | AGGGGTGGATGACCGTGAGT |
| cmo_KG994R_tyr-donor | CTTATTCTTTTCGGAGACACTC |
| cmo_KG882F_tyr_genoyping | TTGTTGGCAAAAGAATGCTG |
| cmo_KG883R_tyr_genotyping | GCTTCATGGGCAAAATCAAT |
| cmo_KG884F_tyr_sequencing | GGATGGGTGATGGGAGTC |
| cmo_KG674F_sox2-v5donor | GCTGCGCCCAGTAGACTGCA |
| cmo_KG675R_sox2-v5donor | TCAGATTTTTCCTACTCTCC |
| cmo_KG823F_sox2F1-genotyping | ACATGATCAGCATGTACCTCC |
| cmo_KG824R_sox2R1-genotyping | TAATTTGGATGGGATTGGTGG |

| **Zebrafish** |  |  |
| --- | --- | --- |
| hey2_F_umi | ctacacgacgctcttccgatctNNWNNVDHACGGCTCGGGGCGTGTTTTCTAT | LAM PCR |
| hey2_R | agacgtgtgctcttccgatctCATCCATTAAGTGAATCATCAGTTCC | Amplification PCR |
| 5p_DS_constant_F | CTACACGACGCTCTTCCGA |  |

| Hey2 avi tag donor | GCTCAGAGAGTGTGTGGTGTCTGTACCTGCGCGCACTGCATCATGTCAGGACTGAACGATATCTTCGAGGCTCAGAAAATCGAGTGGCACGAGGGCGCGCCAAAGCGGCCCTGTGAGGACAGCACGTCCGACAGCGACATGGATGAA |
| --- | --- |
